# Evaluation of the synergistic effects of epigallocatechin-3-gallate-loaded PEGylated-PLGA nanoparticles with nimodipine against neuronal injury after subarachnoid hemorrhage

**DOI:** 10.3389/fnut.2022.953326

**Published:** 2023-01-04

**Authors:** Xianguang Yang, Mengguo Han, Xue Wang, Jian Wang, Xiaoxue Sun, Chunyan Zhang, Shuaiguo Yan, Liyong Huang, Ying Chen

**Affiliations:** ^1^College of Life Sciences, Henan Normal University, Xinxiang, Henan, China; ^2^Department of Neurosurgery, The First Affiliated Hospital of Xinxiang Medical University, Henan, China; ^3^Henan Key Laboratory of Neurorestoratology, The First Affiliated Hospital of Xinxiang Medical University, Henan, China

**Keywords:** EGCG, subarachnoid hemorrhage, PLGA-PEG, nanoparticles, nimodipine, autophagy

## Abstract

Subarachnoid hemorrhage (SAH) is a devastating subtype of stroke with high mortality and morbidity. Although serious side effects might occur, nimodipine, a second-generation 1,4-dihydropyridine calcium channel blocker, is clinically used to improve neurological outcomes after SAH. Recently, (-)-epigallocatechin-3-gallate (EGCG) has been reported to inhibit Ca^2+^ overloading-induced mitochondrial dysfunction, oxidative stress, and neuronal cell death after SAH; however, low bioavailability, instability, and cytotoxicity at a high dose limited the clinical application of EGCG. To overcome these limitations, PEGylated-PLGA EGCG nanoparticles (EGCG-NPs) were constructed to enhance the bioavailability by using the double-emulsion method. Antioxidative activity, cytotoxicity, behavioral, and immunohistochemistry studies were carried out to determine the neuroprotective effectiveness after cotreatment with EGCG-NPs (75 mg/kg/d preconditioning for 7 days before SAH) and nimodipine (10 mg/kg/d after 30 min of SAH) by using *in vivo* SAH models. The optimized EGCG-NPs with a Box–Behnken design showed a small particle size of 167 nm, a zeta potential value of −22.6 mV, an encapsulation efficiency of 86%, and a sustained-release profile up to 8 days *in vitro*. Furthermore, EGCG-NPs (75 mg/kg/d) had superior antioxidative activity to free EGCG (100 mg/kg/d). EGCG-NPs combined with nimodipine exhibited significant synergistic effects against neuronal cell death by suppressing oxidative stress, Ca^2+^ overloading, mitochondrial dysfunction, and autophagy after SAH. These results suggest that cotreatment with EGCG-NPs and nimodipine may serve as a promising novel strategy for the treatment of SAH.

## Introduction

Green tea, the second most popular beverage in the world, is made from fresh leaves of *Camellia sinensis*, which contains abundant polyphenols. Polyphenols have been associated with providing beneficial health effects in the prevention of cancers, diabetes, and neurodegenerative and cardiovascular diseases (1–4). (-)-Epigallocatechin-3-gallate (EGCG) is one of the most abundant compounds, having excellent bioactivities even at low concentrations (plasma levels of ≤10 μM), such as antioxidant and anti-inflammatory properties (5, 6). However, the limitations of EGCG such as its low bioavailability, instability, short half-time, and cytotoxicity at a large dose have hampered its application in clinical settings. Encapsulated EGCG might be effective in overcoming the aforementioned disadvantages, which enhances its stability and increases its concentration in the plasma *via* slow and sustainable release (7). Therefore, several different nanoparticles (NPs) have been formulated, including liposomes, magnetic NPs, and polymeric NPs (8–12). Among these nanocarriers, PLGA is a safety European Medicines Agency (EMA)- and U.S. Food and Drug Administration (FDA)-approved drug carrier with higher biocompatibility and biodegradability. More importantly, PLGA microspheres provide sustained drug release over a period of a few weeks to months. Moreover, PLGA-encapsulated EGCG-NPs exhibited a more therapeutic effect against several cancers, such as non-small-cell lung cancer, breast cancer, and prostate cancer (13, 14). Most recently, loading of EGCG in PEGylated-PLGA NPs (EGCG-NPs) has been found to have neuroprotective effects with no major side effects against epilepsy and Alzheimer's disease, indicating that EGCG-NP administration might be a potential therapeutic strategy in clinical settings (15, 16).

Even though survivors of subarachnoid hemorrhage (SAH) have increased by 17% in the past few decades due to the advanced early diagnosis and interventional therapy, SAH, an uncommon subtype of stroke, remains a leading cause of death and severe disability worldwide (17). It has been reported that multiple factors, including inflammatory responses, oxidative stress, calcium overloading, and mitochondrial dysfunction, are associated with SAH progression and poor outcomes (18). Among them, calcium homeostasis is critical for the physiological maintenance of intra- and extracellular signaling cascades because overloaded calcium triggers secondary ischemia, which results in poor outcomes by inducing the narrowing of intracranial arteries and activating Ca^2+^ signaling pathway-induced cell life-and-death decisions (19). Therefore, blocking calcium channels is a promising strategy to counteract the elevation of intracellular calcium either from the endo- and/or exogenous pool after SAH. Indeed, several calcium antagonists exhibited neuroprotective properties, including nimodipine and nicardipine (20). Nimodipine, an L-type dihydropyridine calcium channel antagonist, is the only FDA-approved drug to improve neurological outcomes and to decrease mortality in patients with SAH (19, 21). The recommended dose of orally administered nimodipine is 30–120 mg every 4 h for 21 days. However, one of the serious side effects of nimodipine is hypoperfusion, which worsens neurological outcomes.

A mutual interplay between calcium signaling pathways and ROS signaling systems might contribute to the pathogenesis of various disorders. In our previous studies, EGCG has been found to act as a voltage-gated calcium channel (VGCC) antagonist by blocking the calcium influx from the extracellular space and as an antioxidative agent by reducing ROS generation (22–24). Indeed, the imbalance between ROS generation and reduction/scavenging inevitably resulted in oxidative stress, which was strongly associated with the diverse pathogenesis of early brain injury (EBI) after SAH. Kang et al. reported that EGCG decreased cell death processes and neurotoxic effects by inhibiting oxidative stress in the hippocampal tissue (25). However, some *in vivo* and *in vitro* pharmacological studies have demonstrated that EGCG at high concentrations exhibited acute and subacute toxicities (26). For example, 50 μM or 50 mg/kg/day of EGCG dramatically increased reactive oxygen species (ROS) generation and exacerbated mitochondria abnormalities (27–29). Several studies have demonstrated the neuroprotective efficacy of EGCG-NPs; however, none of them has been used in *in vivo* SAH therapy. Therefore, the aim of this study was to prepare PEGylated-PLGA EGCG-NPs and investigate its efficacy and safety against SAH. In this study, EGCG-NPs were characterized by particle size and morphology, polydispersity index, drug encapsulation efficiency, and drug loading capacity. The synergetic neuroprotective effects of EGCG-NPs (75 mg/kg/d) with nimodipine (10 mg/kg/d) were evaluated using *in vivo* and *in vitro* SAH models.

## Materials and methods

### Materials

mPEG-poly lactic-co-glycolic acid (PLGA) (75:25, 45,000) was purchased from Daigang Biomaterial Co., Ltd (Jinan, Shandong, China). EGCG (purity > 98%; Yuanye Biotechnology Co., Ltd, Shanghai, China) was dissolved in water (pH 3.0) and stored at −20°C. Nimodipine was obtained from Ruiyang Pharmaceutical Co., Ltd (Yi Yuan, Shandong, China). Lactate dehydrogenase (LDH), glutathione (GSH), superoxide dismutase (SOD), total antioxidant capacity (T-AOC), and malondialdehyde (MDA) kits were obtained from the Nanjing Jiancheng Bioengineering Institute (Nanjing, Jiangsu, China). JC-1, Fluo-3 AM, DCFH-DA, Nissl staining kits, and enhanced chemiluminescence kits were purchased from Beyotime (Shanghai, China). CaMKII, Atg5, Beclin-1, and Mn-SOD antibodies were obtained from Proteintech Group, Inc (Rosemont, IL, USA). SP-9002 SPlink detection and DAB kits were purchased from Zhongshan Golden Bridge Biotechnology (Beijing, China).

### Preparation of EGCG-NPs

EGCG-NPs were prepared by using the double-emulsion method in three steps: (1) EGCG was dissolved in 1 ml of the aqueous phase (W1) at pH 3.0 and emulsified with 1.5 ml of an oil phase (O) composed of ethyl acetate, which contained the dissolved polymeric matrix. Simple emulsion (W1/O) was prepared by using an ultrasound probe at 20% amplitude for 20 s; (2) 2 ml of 2.5% Tween-80 (W2) was added to W1/O, and the mixture was subjected to ultrasound at 20% amplitude for 2 min to yield a double emulsion (W1/O/W2); (3) 2 ml of 0.02% Tween-80 was added dropwise to stabilize the emulsion. Finally, the organic solvent was evaporated by stirring for 24 h.

### Optimization

To obtain the optimal formulation, a 2^3^ Box–Behnken design was used (Table 1). The effect of the independent variables [EGCG (X1), PEG-PLGA (X2), and Tween-80 (X3)] on the dependent variables [average particle size (Zav) (Y1), zeta potential (ZP) (Y2), polydispersity index (PDI) (Y3), and encapsulation efficiency (EE) (Y4)] was evaluated. A total of 15 experiments were required. The response Y was modeled using the following full second-order polynomial equation:


$$\begin{array}{l} \text{Y = }{\text{β}}_{0}+{\text{β}}_{1}{\text{X}}_{1}+{\text{β}}_{2}{\text{X}}_{2}+{\text{β}}_{3}{\text{X}}_{3}+{\text{β}}_{12}{\text{X}}_{1}{\text{X}}_{2}+{\text{β}}_{13}{\text{X}}_{1}{\text{X}}_{3}\\ \;+{\text{β}}_{23}{\text{X}}_{2}{\text{X}}_{3}+{\text{β}}_{11}{\text{X}}_{1}^{2}+{\text{β}}_{22}{\text{X}}_{2}^{2}+{\text{β}}_{33}{\text{X}}_{3}^{2} \end{array}$$

where Y is the established response, β_0_ is the intercept, and β_1_-β_33_ are the measured coefficients computed from the observed experimental values of Y.

**Table 1:** Box-Behnken design used for optimization of EGCG-loaded formulae.

**Independent variables**	**Coded levels**		
	**Low level (−1)**	**Medium level (0)**	**High level (1)**
X_1_: EGCG (mg/ml)	1	5.5	10
X_2_: PEG-PLGA (mg)	12	16	20
X_3_: Tween-80 (%)	1.5	2.25	3
**Dependent variables**	**Goal**		
Y_1_: Average particle size (nm)	Minimize		
Y_2_: Zeta potential (mV)	Maximize in terms of absolute value		
Y_3_: Encapsulation efficiency (%)	Maximize		
Y_4_: Polydispersity index (PDI)	Minimize		

### Physicochemical characterization

The Zav, PDI, and ZP were measured using dynamic light scattering and laser Doppler electrophoresis using an M3 PALS system in Zetasizer Nano ZS (Malvern Instruments, Malvern, UK) at a detection angle of 90°, 25°C with a 5-mW He–Ne laser operating at a 633-nm wavelength. Samples were diluted in water (1:10). The results were the averages of triplicate experiments with an average of 15 runs per measurement.

### Morphology of EGCG-NPs

Transmission electron microscopy (TEM) (JEM 1010, JEOL, Japan) was used to investigate the morphology of the EGCG-NPs. A single drop of the diluted samples (1:10) was mounted on a carbon-coated copper grid, and then negative staining with uranyl acetate (2%) took place to visualize the particles.

### Determination of the encapsulation efficiency

Free EGCG was separated from EGCG-NPs by filtration/centrifugation using an Amicon^®^ Ultra-0.5 centrifugal filter device (Millipore^®^ Co, Massachusetts, USA) for centrifugation at 12,000 × g for 30 min at 4°C. The concentration of EGCG was detected. An Agilent 1100 HPLC system with a UV detector was used for the HPLC analysis by using a C18 analytical column at 40°C. The optimized mobile phase consisted of acetonitrile and buffer. The flow rate was 0.8 ml/min, and the injection volume was set at 15 μl. The UV wavelength was set at 280 nm. Standards with a concentration range of 0.03–0.5 mg/ml were prepared. All experiments were carried out in triplicate. Then, encapsulation efficiency was calculated using the following equation:


$$\begin{array}{l} \text{EE = }(\text{total amount of EGCG}-\text{free amount of EGCG})\\ \;/\text{total amount of EGCG}\times 100\text{\%}. \end{array}$$

### *In vitro* drug release

The release profile of EGCG from the polymeric matrix was determined by bulk equilibrium direct dialysis, as described by Albuquerque et al., with some modifications (30). For this test, two different release mediums were chosen: one medium was composed of an acid buffer solution (pH 3.0) with ascorbic acid 0.25%, and the other medium was PBS 0.1 M buffer solution (pH 7.4) with ascorbic acid 0.25%. The dialysis sacs were equilibrated with the dissolution medium 1 h before the start of the experiment. After that, 5 ml of EGCG-NPs and free EGCG samples were enclosed in dialysis bags (Viskase Inc. MWCO 12–14,000, IL, USA) and then placed in 70 ml of buffers at 37°C with stirring at 50 rpm in a shaking incubator. At predetermined time intervals, 1 ml of the sample was withdrawn from the release medium to be analyzed with HPLC using the conditions described earlier. Then, the same amount of fresh medium was added to a dialysis container. Different kinetic models were used to adjust the data. The assay was carried out in triplicate.

### Animals

The animal use and care protocols were approved by the Institutional Animal Care and Use Committee (IACUC) of Henan Normal University. A total of 60 adult male Kunming mice weighing 18–20 g were purchased from Zhengzhou University. All animals underwent institutional quarantine for 7 days before use. The animals were housed in a temperature-controlled (22 ± 3°C) and humidity-controlled (40–70%) environment with a 12-h light/dark cycle.

#### Experiment 1

To evaluate the antioxidant effect of EGCG-NPs, 24 mice were randomly divided into four groups (*n* = 6): Sham, SAH, SAH + free EGCG (150 mg/kg/d), and SAH + EGCG-NPs (75 mg/kg/d). The samples were collected from serum and ipsilateral hippocampus and cortex, and antioxidative enzymes activities, ROS level and the expression of Mn-SOD were detected. EGCG was intragastrically administered with water for 7 days before OxyHb injection, respectively.

#### Experiment 2

To explore the neuroprotective effects of cotreatment with EGCG-NPs and nimodipine, 30 mice were randomly assigned to five groups (*n* = 6): Sham, SAH, SAH + nimodipine (30 mg/kg), SAH + EGCG-NPs, and SAH + EGCG-NPs+nimodipine (10 mg/kg). Ipsilateral hippocampus and cortex were chosen for immunofluorescence and Nissl staining. Nimodipine was given after 30 min of SAH.

### Mouse SAH model

SAH was induced in mice using the model reported by Huang et al. (31). The animals were placed in a prone position under 1% pentobarbital general anesthesia (50 mg/kg, intraperitoneally). The posterior cervical muscles were dissected through a suboccipital midline skin incision and retracted laterally. The exposed transparent atlantooccipital membrane was penetrated with a 30-gauge needle. Under spontaneous breathing, a 23-gauge needle without a point was percutaneously inserted into the skull at a controlled depth of 1.5 mm in the cross-position at 2 mm from the sagittal suture and 1 mm from the coronal suture. Subsequently, 50 μl (150 μmol/l) of OxyHb was injected through this hole into the subarachnoid space. Then, the animals were euthanized 48 h after SAH. Some were isolated the ipsilateral hippocanpus and cortex and stored at −80°C for enzyme activity assay. The others were perfused with 4% paraformaldehyde in phosphate buffer 0.1 M and then embedded in paraffin. Coronal sections of the brain with the cortex and hippocampus were chosen for the following studies.

### LDH, GSH, SOD, T-AOC, and MDA assay

The LDH, GSH, SOD, T-AOC, and MDA contents in serum and ipsilateral hippocampus and cortex were determined by using specific commercial assay kits according to the manufacturers' instructions.

### Measurement of intracellular reactive oxygen species

To detect the ROS levels of brain tissues, the brain sections were incubated with 10 μM of DCFH-DA at 37°C for 30 min. Excitation and emission wavelengths were set at 485 and 530 nm, respectively. The sections were then observed by fluorescence microscopy (AX10, Zeiss). Fluorescence intensities were quantified by using ImageJ software.

### Cell culture and treatments

PC12 cells were grown in the RPMI 1640 medium with 5% fetal bovine serum (FBS), 10% horse serum, and 1% penicillin/streptomycin at 37°C under 5% CO_2_. At 24 h after seeding the cells, OxyHb was added to a final concentration of 10 μM to establish an *in vitro* SAH model. Then, 25 μm of EGCG-NPs and 10 μm of nimodipine were used.

### Measuring cytosolic Ca^2+^ concentration levels

To investigate the changes in the intracellular calcium level after SAH, the Ca^2+^ concentration was detected using Fluo-3 AM. In brief, 1 × 10^5^ cells were incubated with 2 μM of Fluo-3 AM for 1 h at 37°C in the dark. After gently washing the cells three times with Hanks' balanced salt solution (HBSS), an additional incubation was performed to equilibrate the intracellular dye concentration at room temperature. The fluorescence was continuously monitored at 10-s intervals for up to 8 min using a microplate fluorometer, with excitation wavelength of 506 nm and emission wavelength of 526 nm. The average fluorescence value was presented as relative fluorescence units (RFUs).

### Immunohistochemistry assay

Deparaffinized sections were treated with 0.3% hydrogen peroxide in methanol for 15 min at room temperature to block endogenous peroxidase activity. The sections were incubated in 0.01 M, pH 9.0 EDTA buffer for 20 min at 121°C and cooled to room temperature. After being blocked with 10% normal goat serum for 1 h at room temperature, the sections were subsequently incubated overnight with anti-Mn-SOD, CaMKII, anti-Atg5, and anti-Beclin-1 antibodies. After being extensively washed, the sections were then stained by using the SP-9002 SPlink detection kit. ImageJ software was used to evaluate the expression level.

### Nissl staining

Nissl staining was performed to measure hippocampus injury. After being deparaffinized and hydrated with distilled water, the brain sections were incubated with 0.5% toluidine blue at 37°C for 30 min, then dehydrated with graded alcohols, and cleared in xylene. Finally, these sections were coverslipped using neutral resins and observed under a light microscope by a researcher who was entirely blind to experimental conditions. The neuronal density loss was estimated.

### Neurological score evaluation

The modified Garcia scoring system was blindly assessed for neurological function at 48 h after SAH. The total score ranged from 3 to 18, and the scoring was used based on (1) spontaneous activity, (2) symmetry of limb movement, (3) climbing, (4) body proprioception, (5) the movement of forelimbs, and 6) response to vibrissal touch. Higher scores for the modified Garcia scoring system indicated a better neurological function.

### Statistical analysis

The statistical analysis was performed using the Statistical Package for Social Sciences (SPSS Inc., Chicago, IL) program. All data were reported as means ± SD of three independent experiments. One-way ANOVA, followed by Tukey's *post-hoc* test, was performed for group comparison. The neurological scores were compared using a Kruskal–Wallis non-parametric test, followed by multiple-comparison procedures using Duncan's method. A statistical significance was set at a *P* < 0.05.

## Results

### Optimization study

**Figure 1:**
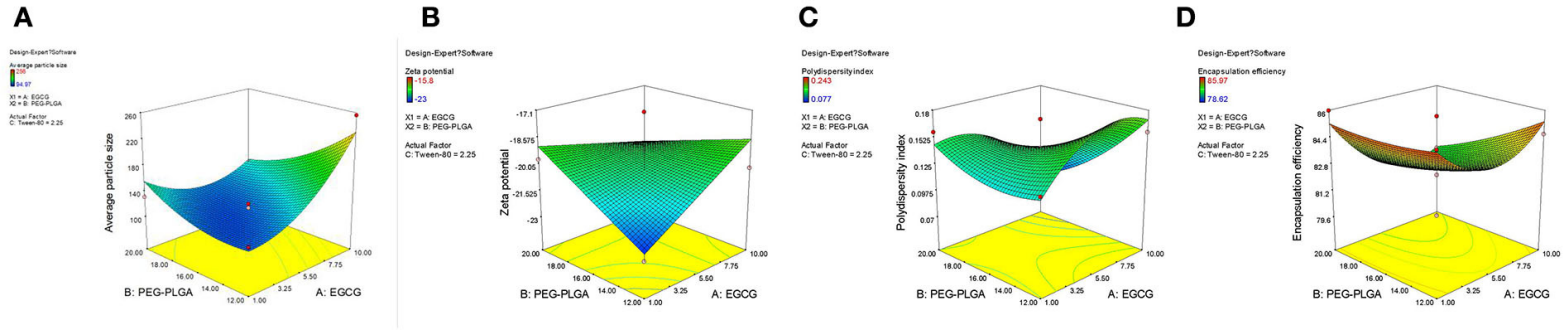
3D surface of the effect of EGCG-NPs. **(A)** Zav surface response at a fixed EGCG concentration of 1.5 mg/ml. **(B)** ZP response at a fixed Tween-80 concentration of 2.25%. **(C)** PDI response. **(D)** EE response at a fixed Tween-80 concentration of 2.25%.

Table 2 shows 15 experimental results of the optimization of EGCG-NPs formulation, with the obtained responses of dependent variables. The particle size of the EGCG-NPs varied from 94.97 to 256 nm and was directly proportional to the concentration of polymer and EGCG (Figure 1A). ZP was found to be in the range of −23 to −15.8 mV, which was inversely proportional to the concentration of polymer and EGCG, indicating that a low dose of EGCG and polymer led to an increase in ZP (Figure 1B). The observed PDI values varied from 0.077 to 0.243, revealing a narrow size distribution and good homogeneity of distribution (Table 2). The most influential parameters of PDI were the concentration of EGCG and polymer, suggesting that the high concentrations of EGCG and polymer induced very broad size distribution of nanoparticles (Figure 1C). The observed EE values varied from 78.62 to 85.97% (Table 2). In contrast to the lower encapsulation efficiency caused by an intermediate concentration of polymer and EGCG-NPs, either a high or a low concentration of polymer and EGCG will increase the encapsulation efficiency, indicating that larger amounts of EGCG can be loaded in the developed NPs (Figure 1D). The multiple regression analysis was performed using the following polynomial equations:


$$\begin{array}{l} {\text{Y}}_{1}=24.36746-20.46965{\text{X}}_{1}+31.52943{\text{X}}_{2}\\ \;+182.20278{\text{X}}_{3}-1.84431{\text{X}}_{1}{\text{X}}_{2}-2.29083{\text{X}}_{1}{\text{X}}_{3}\\ \;-5.39111{\text{X}}_{2}{\text{X}}_{3}+1.02495{\text{X}}_{1}^{2}+1.43811{\text{X}}_{2}^{2}-31.56148{\text{X}}_{3}^{2}\\ {\text{Y}}_{2}=-58.99547+3.68125{\text{X}}_{1}+0.80474{\text{X}}_{2}+4.70611{\text{X}}_{3}\\ \;-0.074583{\text{X}}_{1}{\text{X}}_{2}-0.63083{\text{X}}_{1}{\text{X}}_{3}+0.48889{\text{X}}_{2}{\text{X}}_{3}\\ \;-0.054427{\text{X}}_{1}^{2}-0.05177{\text{X}}_{2}^{2}+0.86519{\text{X}}_{3}^{2}\\ {\text{Y}}_{3}=-0.12365-1.77083{\text{X}}_{1}+0.02815{\text{X}}_{2}+0.19430{\text{X}}_{3}\\ \;-1.500002{\text{X}}_{1}{\text{X}}_{2}-8.83333{\text{X}}_{1}{\text{X}}_{3}+3.7037{\text{X}}_{2}{\text{X}}_{3}\\ \;+7.91667{\text{X}}_{1}^{2}-1.25103{\text{X}}_{2}^{2}-0.01125{\text{X}}_{3}^{2}\\ {\text{Y}}_{4}=110.24262-1.72972{\text{X}}_{1}+0.097531{\text{X}}_{2}-11.60481{\text{X}}_{3}\\ \;-0.041528{\text{X}}_{1}{\text{X}}_{2}+0.56417{\text{X}}_{1}{\text{X}}_{3}-0.25852{\text{X}}_{2}{\text{X}}_{3}\\ \;+0.014609{\text{X}}_{1}^{2}+0.087346{\text{X}}_{2}^{2}+1.11778{\text{X}}_{3}^{2}. \end{array}$$

**Table 2:** Box-Behnken design matrix with values and the measured response.

**Run**	**X_1_**	**X_2_**	**X_3_**	**Y_1_**	**Y_2_**	**PDI**	**Y_3_**
1	0	0	0	111.07 ± 1.69	−20.47 ± 1.17	0.142 ± 0.02	79.66 ± 1.23
2	1	1	0	135.87 ± 1.95	−20.37 ± 0.78	0.077 ± 0.01	81.89 ± 2.01
3	0	−1	−1	121.07 ± 4.40	−20.87 ± 0.35	0.132 ± 0.04	84.7 ± 0.87
4	−1	−1	0	118.47 ± 2.41	−23 ± 0.43	0.131 ± 0.04	85.6 ± 1.34
5	0	0	0	119.97 ± 1.95	−20.6 ± 1.30	0.118 ± 0.04	82.17 ± 0.79
6	−1	0	−1	113.83 ± 5.02	−22.8 ± 1.31	0.103 ± 0.07	84.42 ± 0.96
7	0	1	−1	189.1 ± 2.55	−21.7 ± 0.78	0.111 ± 0.04	85.65 ± 1.52
8	−1	1	0	256 ± 4.80	−20.23 ± 0.31	0.158 ± 0.02	84.61 ± 1.68
9	1	−1	0	131.13 ± 4.33	−19.77 ± 1.00	0.158 ± 0.05	85.97 ± 1.07
10	1	0	−1	133.8 ± 5.05	−17.8 ± 0.46	0.11 ± 0.03	78.62 ± 0.82
11	−1	0	1	106.93 ± 3.47	−18.03 ± 0.58	0.243 ± 0.07	84.7 ± 2.54
12	0	1	1	94.97 ± 0.28	−15.8 ± 0.95	0.117 ± 0.05	83.33 ± 2.17
13	1	0	1	99.41 ± 1.31	−20.6 ± 0.79	0.144 ± 0.04	85.67 ± 1.62
14	0	0	0	113.5 ± 0.56	−17.2 ± 0.17	0.171 ± 0.01	85.64 ± 0.77
15	0	−1	1	99.72 ± 0.86	−21.57 ± 1.41	0.088 ± 0.04	85.87 ± 1.46

X1, X2, X3, Y1, Y2, Y3, Y4. Y_1_, Average particle size (nm); Y_2_, Zeta potential (mV); Y_3_, Encapsulation efficiency (%); Y_4_, Polydispersity index (PDI).

### Validation of optimized EGCG-NPs formulation

**Figure 2:**
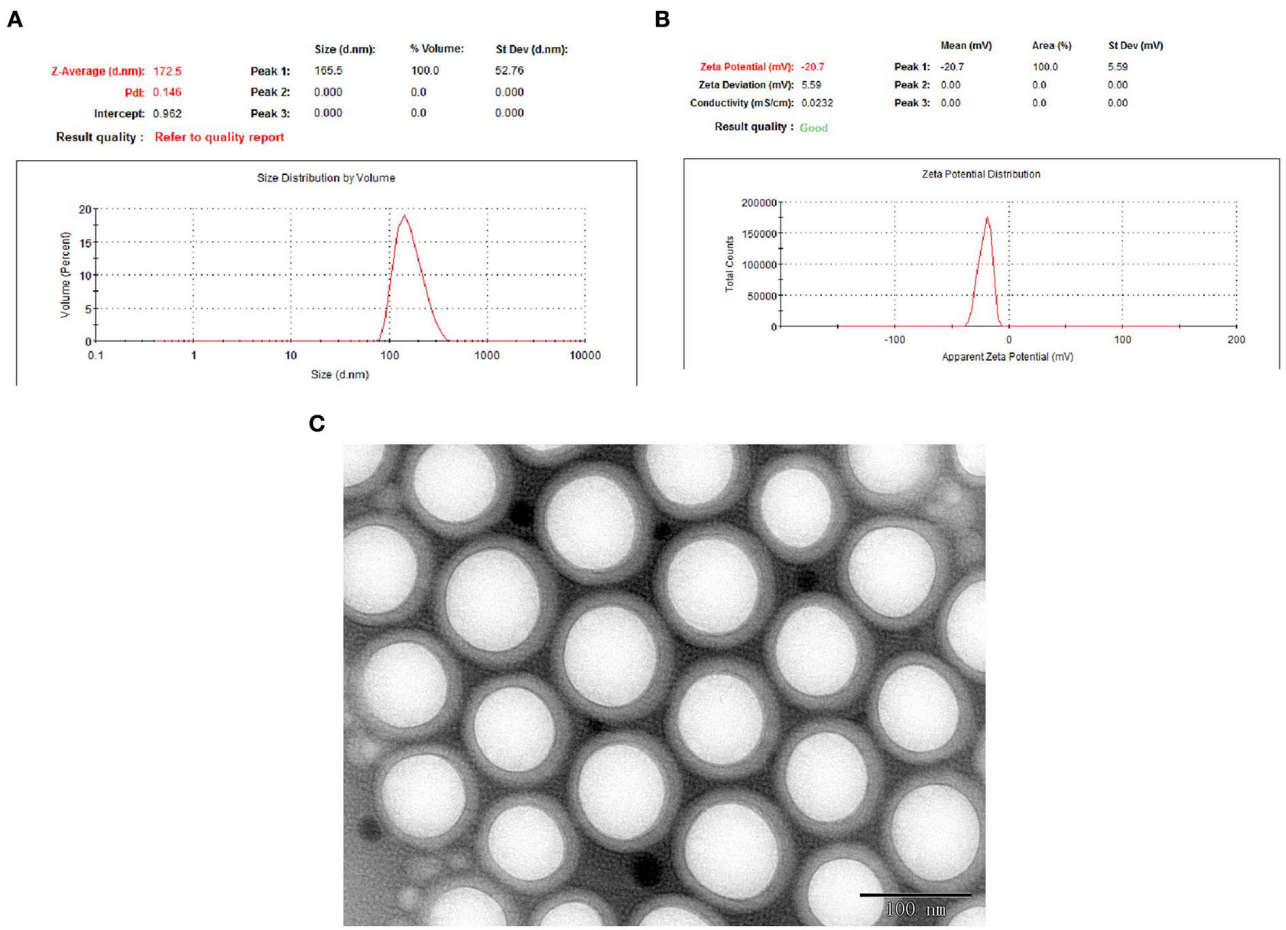
Optimization of EGCG-NPs. Particle size distribution of EGCG-NPs measured with electrophoretic light-scattering (ELS) analyzer **(A)**. Zeta potential measurement of EGCG-NPs **(B)**. TEM image of EGCG-NPs **(C)**. Scale bar = 100 nm.

To promote drug release from the polymeric matrix, a high drug-loading formulation with low-surfactant level was used to develop nanoparticles. Based on the Box–Behnken design, the optimal formulation with 7.0 mg/ml of EGCG, 12 mg/ml of PLGA-PEG, and Tween-80 1.2% was obtained. The optimized formulation was composed of 167 nm Zav, −22.6 mV ZP, 86% EE, and 0.136 PDI (Figures 2A, B). The morphology of the optimized EGCG-NPs formulation was observed using TEM, with the microspheres showing no aggregation (Figure 2C). The diameter of the EGCG-NPs was consistent with the results obtained from an electrophoretic light-scattering spectrophotometer experiment. PEG located toward the aqueous phase creates a smooth surface and a hydrophilic layer surrounding the particles, which increases the stability of the sample.

### *In vitro* release of EGCG-NPs

**Figure 3:**
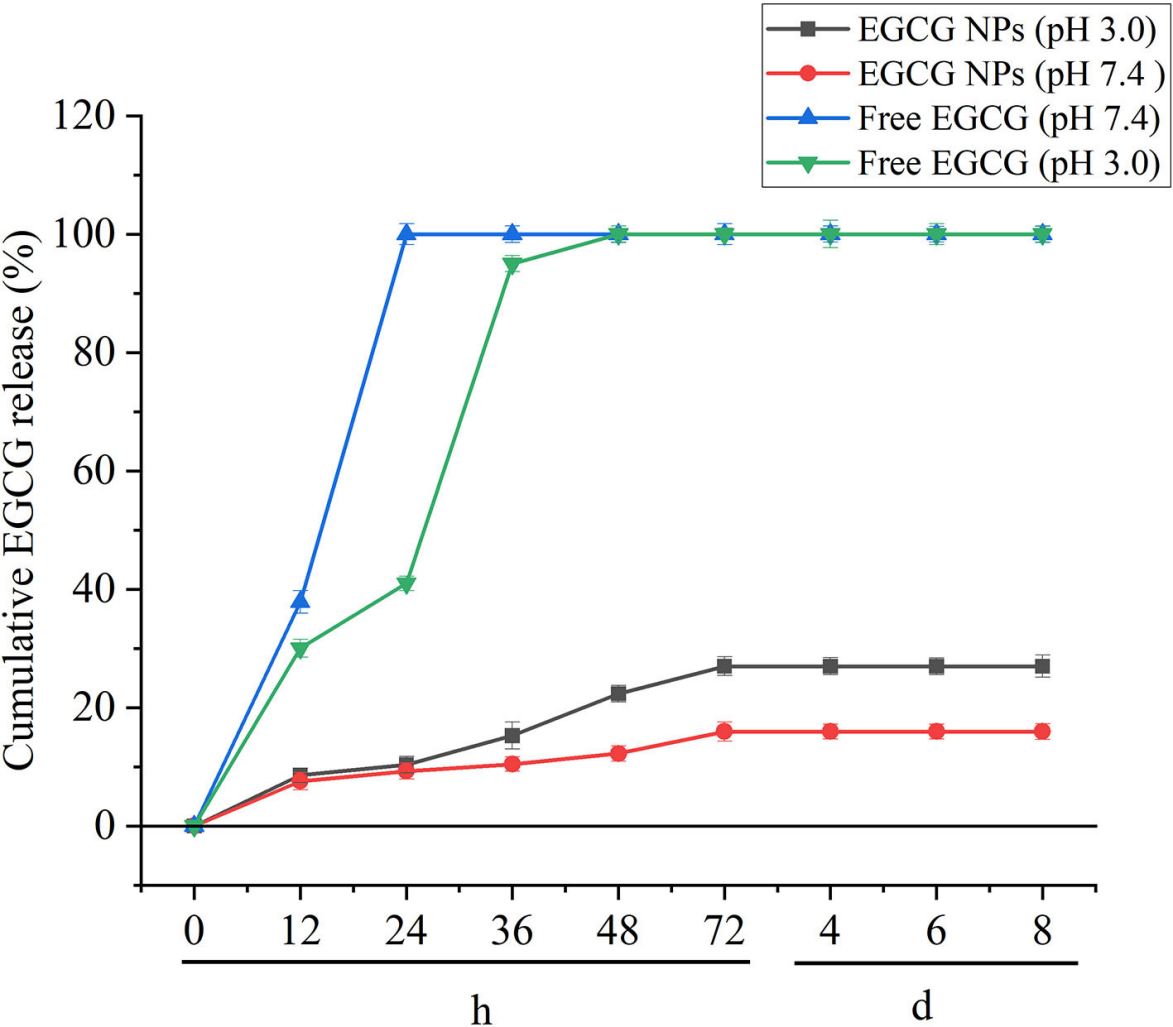
*In vitro* EGCG release profile of free EGCG and EGCG-NPs in pH 3.0 and pH 7.4 buffers. Data are expressed as mean ± SD (*n* = 3).

The diffusion/degradation process of the polymer matrix is the limited step to control the encapsulated drugs released from the polymeric delivery systems (32). *In vitro* release tests were performed to investigate the EGCG-NPs release profile on pH 3.0 and pH 7.0 medium (Figure 3). EGCG showed an initial burst (in acid buffer and in PBS) owing to the superficially entrapped drug. Free EGCG showed faster kinetics than EGCG-NPs. After 6 h, more than 90% of free drugs were released, whereas the released EGCG-NPs were ~10% of the initial amount. The best fit with the highest correlation was found for the hyperbola model for free EGCG, EGCG-NPs in acid buffer, and EGCG-NPs in PBS, indicating a diffusion-governed release.

### EGCG-NPs inhibition OxyHb-induced oxidative stress

**Figure 4:**
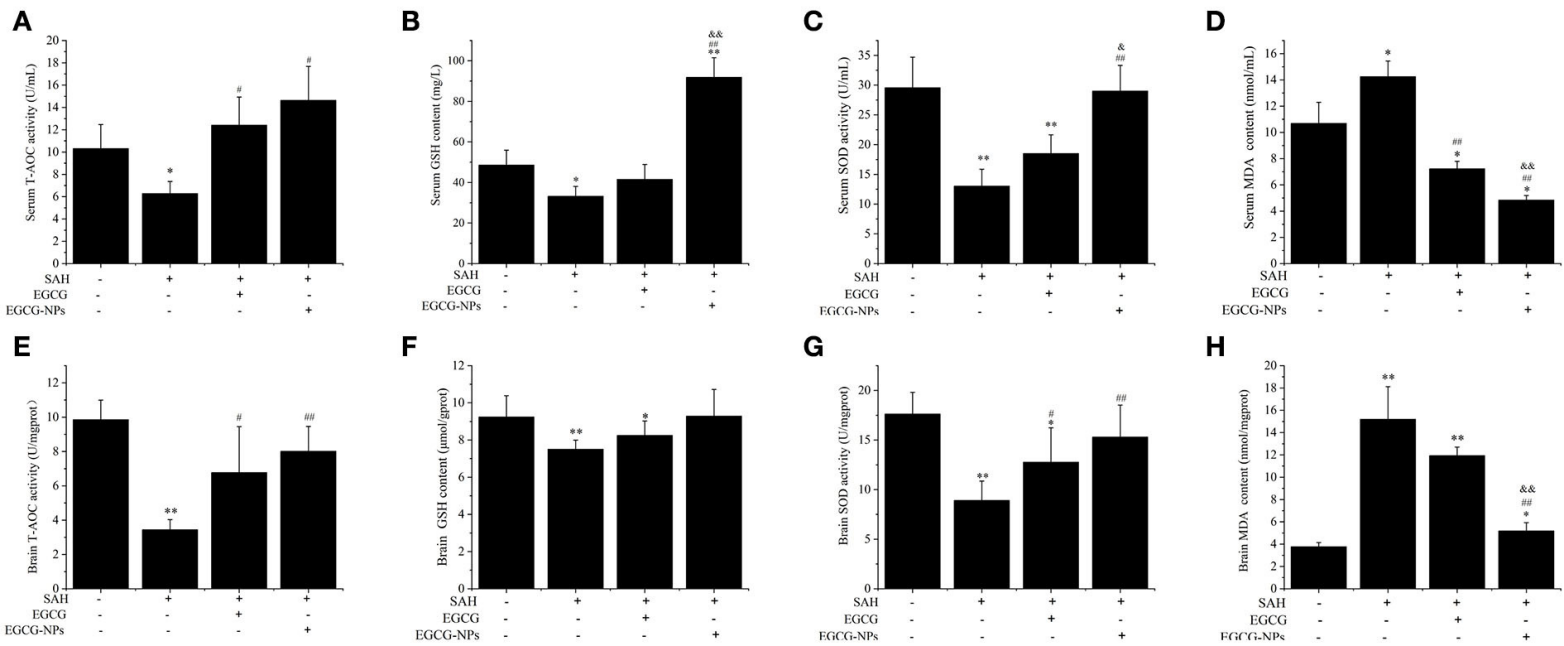
Effects of EGCG-NPs on the regulation of antioxidative enzymes activities after SAH. Serum T-AOC, GSH, SOD activities, and MDA level, respectively **(A–D)**. T-AOC, GSH, SOD activities, and MDA levels in the brain, respectively **(E–H)**. Values represent three independent experiments. *P < 0.05 vs. Sham, **P < 0.01 vs. Sham; ^#^*P* < 0.05 vs. SAH, ^##^*P* < 0.01 vs. SAH; ^&^*P* < 0.05 vs. EGCG, ^&&^*P* < 0.01 vs. EGCG.

To determine whether EGCG-NPs have stronger antioxidative activity than free EGCG, we examined the T-AOC, GSH, SOD, and MDA after SAH. As shown in Figures 4A–D, the lowest serum level of T-AOC, GSH, and SOD in the SAH group (*P* < 0.05 vs. Sham) was restored by both EGCG and EGCG-NPs groups (*P* < 0.05 vs. SAH). However, T-AOC activity reached its highest peak after EGCG-NPs treatment, but there was no significant difference between EGCG and EGCG-NPs groups. In contrast to the EGCG-induced upregulation of GSH and SOD, EGCG-NPs enhanced the GSH and SOD (*P* < 0.01 vs. Sham; *P* < 0.01 vs. SAH) activities and showed a significant difference compared with the EGCG group (*P* < 0.01 vs. EGCG; *P* < 0.05 vs. EGCG). As a result, the serum MDA level was significantly downregulated by EGCG-NPs (*P* < 0.01 vs. EGCG). Similar to the serum GSH, T-AOC, SOD, and MDA levels, the activities of antioxidative enzymes in ipsilateral hippocampus and cortex were higher in the EGCG-NPs group than in the EGCG group (Figures 4E–H).

### EGCG-NPs reduced ROS generation by increasing Mn-SOD activity after SAH

**Figure 5:**
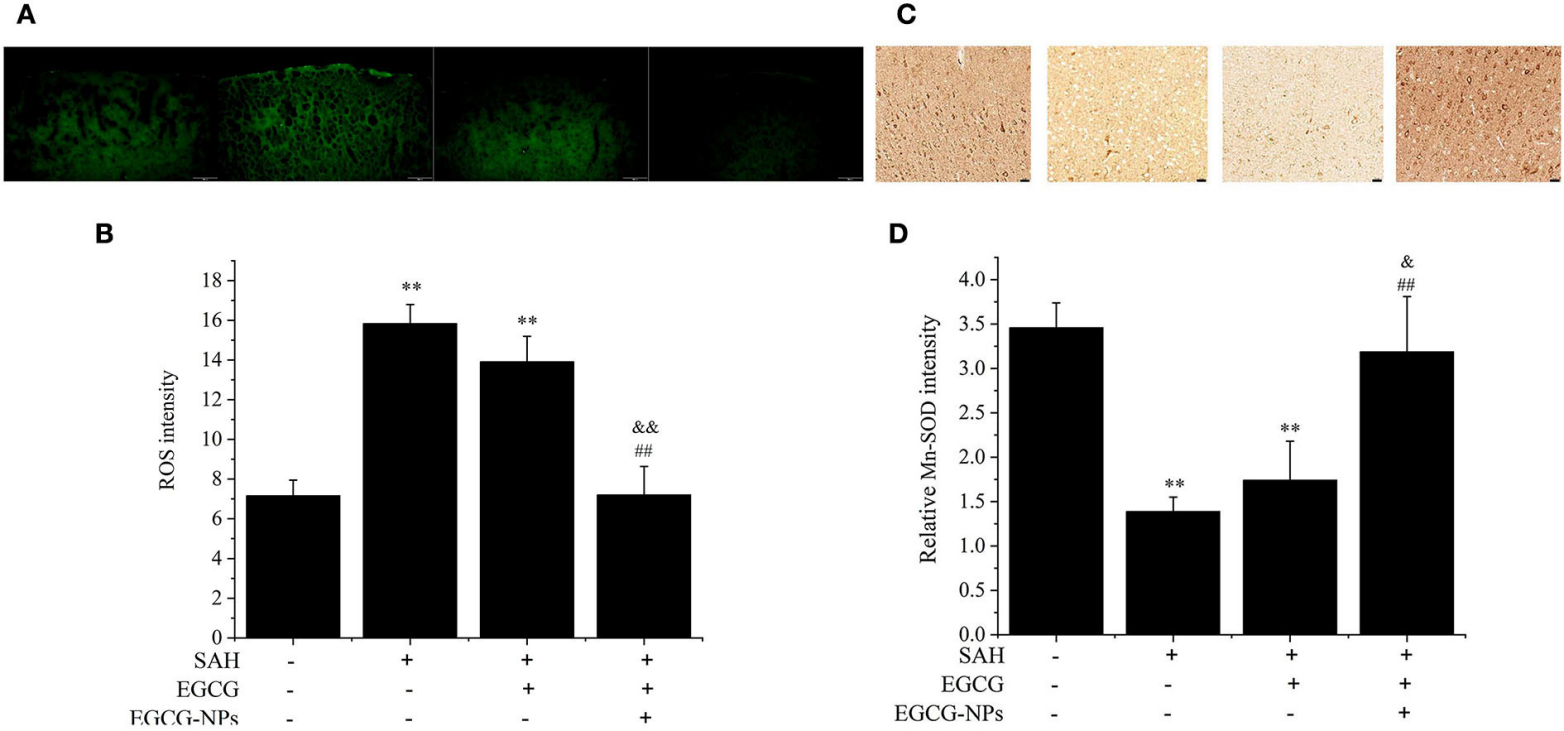
Effects of EGCG-NPs on the mitochondrial oxidative stress after SAH. ROS fluorescence distribution in the brain detected by fluorescence microscopy **(A)**. Quantification of ROS fluorescence intensity after SAH (scale bar = 100 μm) **(B)**. Identification of the expression of Mn-SOD by the immunohistochemical assay (scale bar = 20 μm) **(C)**. Quantification of the Mn-SOD level by densitometry **(D)**. Quantification represents the means and standard deviations of the results from three independent experiments. **P < 0.01 vs. Sham; ^##^*P* < 0.01 vs. SAH; ^&^*P* < 0.05 vs. EGCG; ^&&^*P* < 0.01 vs. EGCG.

In order to investigate the mitochondrial function after EGCG-NPs treatment, we measured the changes in intracellular ROS and Mn-SOD levels. OxyHb exposure significantly increased the intracellular ROS level (*P* < 0.01 vs. Sham), whereas preconditioning EGCG and EGCG-NPs decreased the ROS level (*P* < 0.01 vs. SAH) (Figures 5A, B). Notably, there is a significant difference between these two groups (*P* < 0.01 vs. EGCG). Considering that Mn-SOD is a major player in scavenging ROS in mitochondria, we next detected Mn-SOD expression (Figures 5C, D). Consistent with the ROS level, the low expression of Mn-SOD in SAH and EGCG groups was significantly increased in the EGCG-NPs group (*P* < 0.01 vs. SAH, *P* < 0.01 vs. EGCG), suggesting that EGCG-NPs downregulated ROS generation after SAH mainly by maintaining the mitochondrial function.

### Cotreatment with EGCG-NPs and nimodipine inhibition Ca^2+^ overloading and CaMKII activation after SAH

**Figure 6:**
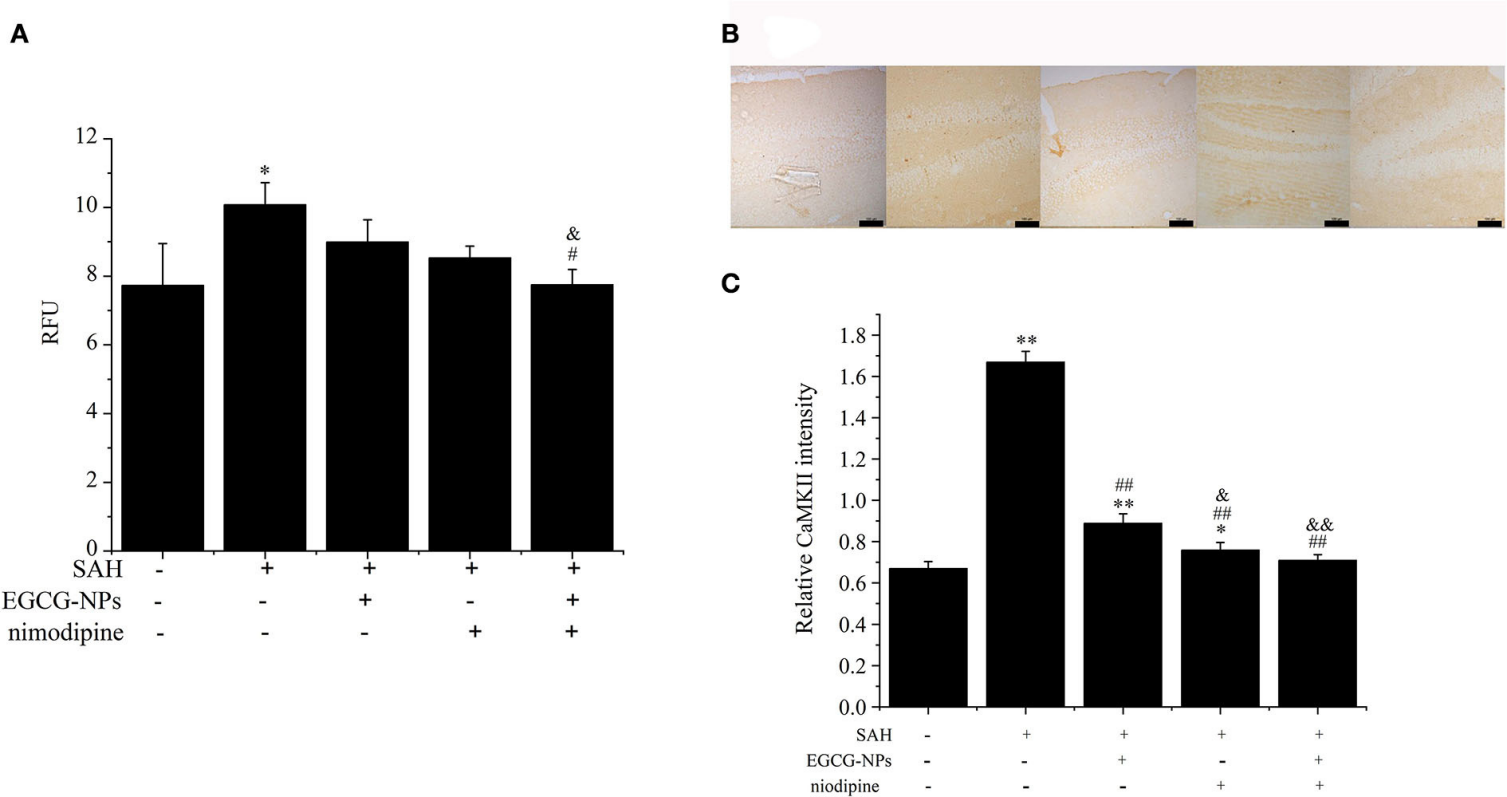
Cotreatment of EGCG-NPs and nimodipine attenuated the intracellular Ca^2+^ levels and the expression of CaMKII after SAH. EGCG-NPs + nimodipine significantly attenuated the Ca^2+^ overloading after SAH **(A)**. Fluorescence was monitored at a measurement frequency of 10 s using a fluorescence plate reader. Cotreatment of EGCG-NPs and nimodipine inhibited the expression of CaMKII **(B)**, scale bar = 100 μm. Quantification of the CaMKII level by densitometry **(C)**. Scale bar = 100 μm. *P < 0.05 vs. Sham, ** *P* < 0.01 vs. Sham; ^#^*P* < 0.05 vs. SAH, ^##^*P* < 0.01 vs. SAH; ^&^*P* < 0.05 vs. EGCG, ^&&^*P* < 0.01 vs. EGCG.

Previous studies have demonstrated that Ca^2+^ overloading is a major player inducing secondary brain injury. To determine the effects of EGCG-NPs and nimodipine on Ca^2+^ concentration after SAH, the cytosolic Ca^2+^ level was measured using Fluo-3 AM. A high Ca^2+^ level in the SAH group can be downregulated by preconditioning EGCG-NPs and nimodipine, but there is no significant difference between these two groups (*P* < 0.05 vs. Sham). However, EGCG-NPs + nimodipine showed stronger effects on the regulation of calcium homeostasis than EGCG-NPs or nimodipine, and a significant difference was observed (*P* < 0.01 vs. SAH; *P* < 0.05 vs. EGCG; *P* < 0.01 vs. nimodipine) (Figure 6A).

Calcium overloading is an important pathological event in SAH development, which activates the CaMKII-mediated signaling pathway. In this study, the expression of CaMKII was markedly activated after SAH, but EGCG-NPs, nimodipine, and cotreatment with EGCG-NPs and nimodipine significantly inhibited the expression of CaMKII (*P* < 0.01 vs. SAH) (Figures 6B, C). Compared with EGCG-NPs, both nimodipine and EGCG-NPs + nimodipine dramatically inhibited the expression of CaMKII (*P* < 0.05 vs. EGCG, *P* < 0.01 vs. EGCG, respectively), but there was no statistically significant difference between these two groups.

### EGCG-NPs synergistic nimodipine reduction autophagy after SAH

**Figure 7:**
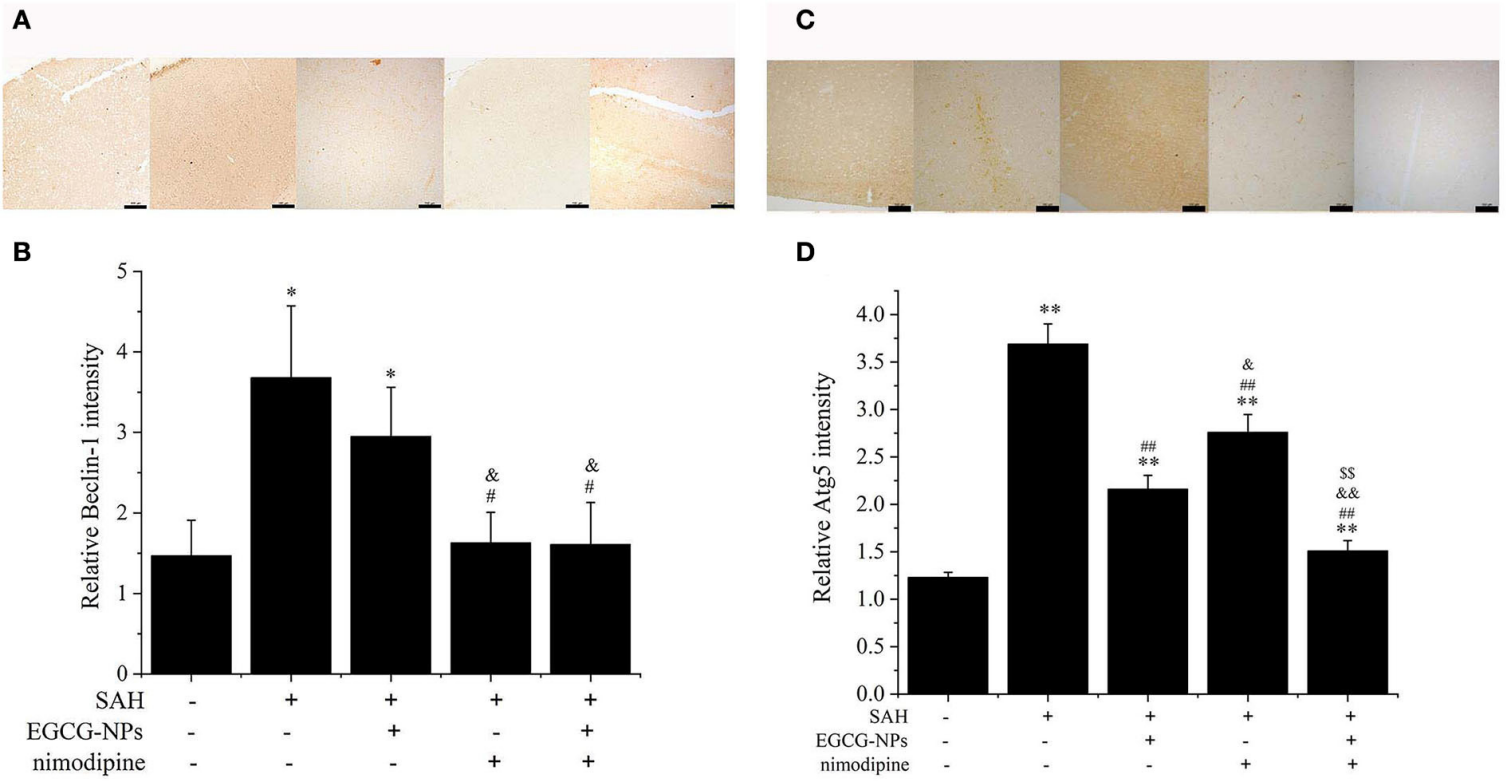
Cotreatment of EGCG-NPs and nimodipine inhibited autophagy induced by OxyHb. Identification of the expression of Beclin-1, scale bar = 100 μm **(A)**. Quantification of the expression of Beclin-1 **(B)**. Immunohistochemical microscope image of Atg5, scale bar = 100 μm **(C)**. Quantification of the Atg5 level by densitometry **(D)**. *P < 0.05 vs. Sham, **P < 0.01 vs. Sham; ^#^*P* < 0.05 vs. SAH, ^##^*P* < 0.01 vs. SAH; ^&^*P* < 0.05 vs. EGCG, ^&&^ *P* < 0.01 vs. EGCG; ^$$^*P* < 0.01 vs. nimodipine.

Activated CaMKII can directly phosphorylate Beclin-1, activating autophagy, which is the main pathway for eliminating abnormal mitochondria. As shown in Figure 7A, the expression of Beclin-1 was similar to that of CaMKII. In normal brain tissue, faint Beclin-1 staining signals were observed, whereas strong Beclin-1 staining signals were found in the SAH group (*P* < 0.01 vs. Sham). The moderate Beclin-1 was only observed in the EGCG-NPs group, but not in the nimodipine and EGCG-NPs + nimodipine group, indicating that the expression of Beclin-1 largely relied on the CaMKII activation (Figure 7B). As for Atg5, the strongest staining signals were detected in the SAH group (*P* < 0.01 vs. Sham). The expression of Atg5 can be inhibited in the other three groups (*P* < 0.01 vs. SAH), but faint signals were observed in EGCG-NPs+ nimodipine group (*P* < 0.01 vs. EGCG; *P* < 0.01 vs. nimodipine) (Figures 7C, D).

### Synergistic effects of EGCG-NPs and nimodipine improve the pathological changes after SAH

**Figure 8:**
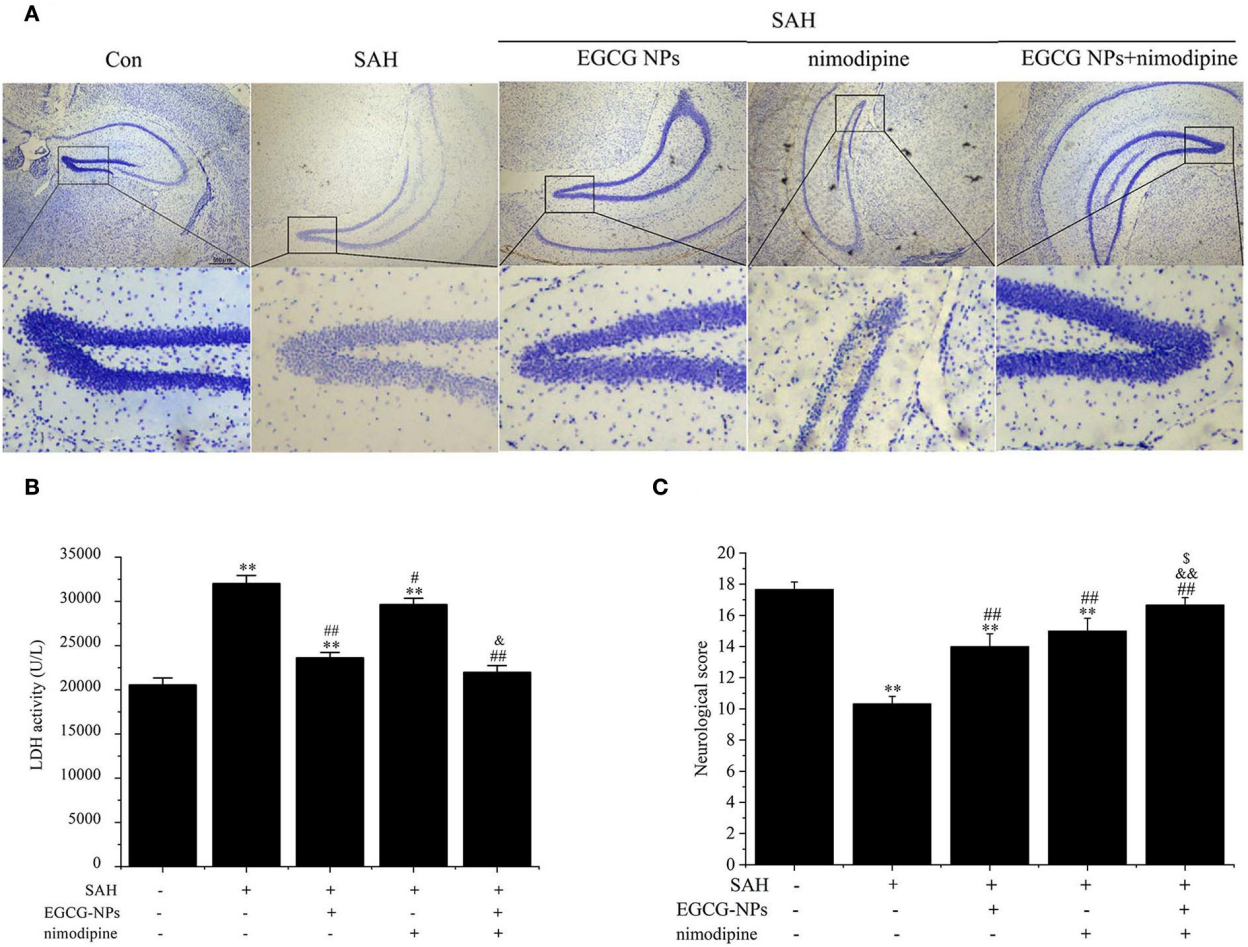
Cotreatment of EGCG-NPs and nimodipine significantly improves the pathological changes after SAH. Representative micrographs of Nissl staining in the hippocampal DG region, scale bar = 500 μm **(A)**. LDH levels after SAH **(B)**. Neurological deficits after SAH **(C)**. Results are presented as the means and standard deviations of the results of three independent experiments. **P < 0.01 vs. Sham; ^#^*P* < 0.05 vs. SAH, ^##^*P* < 0.01 vs. SAH; ^&^*P* < 0.05 vs. EGCG, ^&&^*P* < 0.01 vs. EGCG; ^$^*P* < 0.05 vs. nimodipine.

Nissl staining also showed that both EGCG-NPs and nimodipine treatment groups showed an obvious reverse to the severe damage in the dentate gyrus areas of the hippocampus after SAH (Figure 8A). However, there was significantly less neuron loss and shrinkage morphology of neurons in cotreatment with EGCG-NPs and nimodipine groups than those in either EGCG-NPs or nimodipine group.

The neuroprotective role of EGCG-NPs was also evaluated by detecting the LDH level, which was widely regarded as a cell injury marker after SAH (Figure 8B). The LDH level was significantly increased after SAH (*P* < 0.01 vs. Sham), whereas treatment with EGCG-NPs and nimodipine significantly downregulated LDH activity. Specifically, treatment with EGCG-NPs led to a lower LDH activity than treatment with the nimodipine group (*P* < 0.01 vs. EGCG-NPs). However, the lowest LDH level was observed in cotreatment with EGCG and nimodipine groups (*P* < 0.01 vs. Sham, *P* < 0.01 vs. EGCG-NPs, *P* < 0.01 vs. nimodipine), which almost reached the normal level.

Similar to the LDH and Nissl staining results, the neurological scores, as shown in Figure 8C, were significantly lower 48 h after SAH than those of the Sham group (*P* < 0.01 vs. Sham). However, both EGCG-NPs and nimodipine improved neurological scores, with no significant difference between these two groups (*P* < 0.01 vs. Sham; *P* < 0.01 vs. SAH). Cotreatment with EGCG-NPs and nimodipine considerably improved the neurological performance, showing a statistically significant difference with either EGCG-NPs or nimodipine (*P* < 0.01 vs. Sham; *P* < 0.01 vs. SAH; *P* < 0.01 vs. EGCG-NPs; *P* < 0.05 vs. nimodipine), indicating that impaired behavior function caused by SAH can be restored by EGCG-NPs + nimodipine.

## Discussion

A growing body of evidence supports a correlation between EGCG intake and a reduced risk of central nervous diseases (33, 34). Recently, we have reported the efficacy of EGCG in SAH treatment by inhibiting calcium-induced autophagy (22–24). This study describes EGCG-NPs as a novel therapeutic approach for the treatment of SAH, including the following novel findings: (1) PEGylated-PLGA-loaded EGCG-NPs were successfully synthesized; (2) EGCG-NPs exhibited stronger antioxidative stress activity than free EGCG; (3) cotreatment with EGCG-NPs and nimodipine inhibited Ca^2+^ overloading and CaMKII activity; (4) cotreatment with EGCG-NPs and nimodipine eliminated dysfunctional mitochondria by autophagy; and (5) EGCG-NPs + nimodipine significantly reversed histopathological alterations after SAH.

Even though free EGCG has been found to exhibit antioxidative activities and mitochondrial protective effects after SAH, its poor bioavailability, low stability, and potential toxicity restricted its usage in clinical settings. Therefore, a nanoparticle formulation based on the biodegradable PLGA polymer was performed. Previous research studies have synthesized PEGylated-PLGA-loaded EGCG-NPs, showing an increase in the penetrating capability of the blood–brain barrier (BBB). However, low drug loading restricted its use both in the laboratory and clinical studies (10). In this study, 7 mg of EGCG was used to synthesize EGCG-NPs, and highly monodisperse and uniform EGCG-NPs were spherical in shape and had an average diameter of 167 nm, a PDI value of 0.136, a zeta potential value of −22.6 mV, and an EE of 86%. Particles smaller than 200 nm and the surfactant (Tween-80) might enhance the penetration of EGCG-NPs through the BBB. In addition, a suitable release of the drug from PLGA resulted in a prolonged permanence time in blood. Polymeric surface modification with PEG has also been demonstrated to enhance the rapid spread of carried drugs within brain tissue by reducing the reticuloendothelial system uptake (35). As a result, synthesized EGCG-NPs might exhibit stronger bioavailability and stability than free EGCG. These findings are consistent with the present study showing that the low concentration of EGCG-NPs (75 mg/kg) had stronger antioxidative properties than free EGCG (150 mg/kg) in the treatment of SAH, that is, upregulation of GSH, SOD, and T-AOC activities and downregulation of MDA and ROS levels.

ROS are not only harmful oxidative stressors but also function as well-accepted second messengers by regulating target proteins in a variety of physiological and pathological conditions, as well as in SAH progression. ROS modulates plasma membrane/intracellular Ca^2+^ channels and Ca^2+^ ATPase activities to maintain intracellular calcium homeostasis (36). Calcium signals, in turn, are essential for the formation of free radicals because an elevated intracellular Ca^2+^ level regulates ROS-generating enzymes (37). Considering that EGCG-NPs inhibited ROS generation after SAH, the intracellular Ca^2+^ level was detected. The level of Ca^2+^ concentration in cotreatment with EGCG-NPs and nimodipine groups was significantly lower than that in either EGCG-NPs or nimodipine group. In recent years, substantial antagonists/agonists have been chosen to block or activate one signaling pathway but, finally, failed to be successfully used in the clinic, such as NO donors, calcium channel blockers, antioxidants, and anti-inflammatory agents. Subsequently, some researchers proposed that the combination of two or multiple treatments may improve the SAH outcome due to the cross-talk between pathological pathways. In our previous studies, EGCG has been reported to reduce Ca^2+^ overloading, reduce ROS generation, and eventually prevent mitochondrial function and neuronal cell death. Therefore, EGCG combined with nimodipine might be a novel strategy in the treatment of SAH by targeting ROS and calcium signaling pathways. Due to the pivotal roles of mitochondria in regulating ROS generation, Ca^2+^ homeostasis, and cell growth and differentiation, a growing body of studies is focusing on developing strategies to precisely maintain sustainable mitochondrial function. In line with previous studies, cotreatment with EGCG-NPs significantly reduced mitochondrial oxidative stress than free EGCG, indicating that EGCG-NPs might directly protect mitochondrial function after SAH.

The accumulation of dysfunctional mitochondria was deleterious and caused serious consequences, even led to cell death. Autophagy plays pivotal roles in cellular quality control by alleviating abnormal mitochondria; therefore, moderate activation of autophagy predominantly acts as a pro-survival pathway after SAH (38–41). On the other hand, either defective or excess autophagy can promote cell injury and death after SAH (42–44). CaMKII, the major calcium-dependent signaling protein, has been evoked either by intracellular Ca^2+^ overloading or by ROS (45–47). Recently, CaMKII has been reported to directly phosphorylate Beclin-1 at Ser90, resulting in the ubiquitination of Beclin-1 and initiation of autophagy (48). Therefore, the CaMKII signaling pathway might be a potential target in SAH therapy (49–51). Bensalem et al. have demonstrated that polyphenols upregulated the expression of CaMKII to inhibit a cognitive decline in middle-aged mice (52). Green tea polyphenols also exhibited a similar bioactivity profile by upregulation of the expression of CaMKII to attenuate cognitive deficits (53). In contrast to these findings, our study showed that the over-expression of CaMKII activated autophagy through Beclin-1 and Atg5 in SAH, whereas cotreatment with EGCG-NPs and nimodipine downregulated autophagy by maintaining calcium homeostasis, indicating that EGCG-NPs synergistically improved the neuroprotective effects of nimodipine by regulating mitochondrial function, ROS generation, and antioxidative enzyme activation and eventually protecting neuronal cells against OxyHb insult.

## Conclusion

Results of this study confirmed that the EGCG-NP formulation represents a promising drug delivery strategy to enhance its antioxidative activity and related mitochondrial protection. The findings clearly indicated that cotreatment with EGCG-NPs and nimodipine exhibited stronger neuroprotective effects by maintaining calcium homeostasis, mitochondrial function, and autophagy flux. Taken together, EGCG-NPs combined with nimodipine can be regarded as a potential novel pharmacological strategy in SAH.

## Data availability statement

The original contributions presented in the study are included in the article/supplementary material, further inquiries can be directed to the corresponding authors.

## Ethics statement

The animal study was reviewed and approved by Institutional Animal Care and Use Committee (IACUC) of Henan Normal University.

## Author contributions

XY, YC, and LH designed the study. XS, MH, and JW performed the experiments. XW, CZ, and MH performed data collection. XS, CZ, SY, and JW performed statistical analyses. XY, MH, LH, and YC wrote the paper. All authors contributed to the article and approved the submitted version.
